# Fluid dynamics and cell‐bound Psl polysaccharide allows microplastic capture, aggregation and subsequent sedimentation by *Pseudomonas aeruginosa* in water

**DOI:** 10.1111/1462-2920.15916

**Published:** 2022-02-02

**Authors:** Manuel Romero, Alessandro Carabelli, Michael R. Swift, Michael I. Smith

**Affiliations:** ^1^ National Biofilms Innovation Centre, Biodiscovery Institute and School of Life Sciences University of Nottingham Nottingham UK; ^2^ Department of Medicine University of Cambridge Cambridge UK; ^3^ School of Physics and Astronomy University of Nottingham Nottingham NG7 2RD UK

## Abstract

Decades after incorporating plastics into consumer markets, research shows that these polymers have spread worldwide. Fragmentation of large debris leads to smaller particles, collectively called microplastics (MPs), which have become ubiquitous in aquatic environments. A fundamental aspect of understanding the implications of MP contamination on ecosystems is resolving the complex interactions of these artificial substrates with microbial cells. Using polystyrene microparticles as model polymers, we conducted an exploratory study where these interactions are quantitatively analyzed using an *in vitro* system consisting of single‐bacterial species capturing and aggregating MPs in water. Here we show that the production of Psl exopolysaccharide by *Pseudomonas aeruginosa* (PA) does not alter MPs colloidal stability but plays a key role in microspheres adhesion to the cell surface. Further aggregation of MPs by PA cells depends on bacterial mobility and the presence of sufficient flow to prevent rapid sedimentation of early MP‐PA assembles. Surprisingly, cells in MP‐PA aggregates are not in a sessile state despite the production of Psl, enhancing the motility of the aggregates by an order of magnitude relative to passive diffusion. The generated data could inform the creation of predictive models that accurately describe the dynamics and influence of bacterial growth on plastics debris.

## Introduction

The extensive use and inadequate disposal of plastics in modern life, combined with the long persistence of these synthetic polymers in the environment, has resulted in plastic debris being recognized as a serious threat to human health and ecosystems (Kvale *et al*., 2021; Rahman *et al*., 2021). Consequently, plastics as pollutants have become an important area of study, especially in aquatic habitats where many studies focus on the properties, prevalence and role of microplastics (MPs) in contaminating these environments. Microplastics are defined as particles smaller than 5 mm and derived from the weathering of larger plastic pieces or explicitly fabricated small‐scale plastics (Thompson *et al*., 2004). The most frequently identified plastics in the environment are petroleum‐based synthetic plastics such as polyethylene, polyvinyl chloride, polypropylene, polyethylene terephthalate and polystyrene (PS) (Rochman *et al*., 2013).

A fundamental aspect to understand the implications of MP contamination on ecosystems is to resolve the complex interactions of these artificial substrates with microbial cells. In aquatic environments, the transport of MPs in the water systems will be strongly influenced by its modified buoyancy due to association with microorganisms, with important implications on the distribution of MPs (Long *et al*., 2015; Kooi *et al*., 2017). Another potential outcome of MPs accumulation in microbial biofilms is plastic degradation, as MPs could be surrounded by other organic matter and enzymes that may accelerate chemical and physical plastic dissolving processes. Moreover, since microbial communities are the primary niches for nutrient and carbon transformations and form the base of food webs in nature, MP‐associated biofilm communities are expected to influence the biodegradability of these pollutants (Rummel *et al*., 2017). Indeed, recent studies have unveiled several groups of bacteria and fungi capable of degrading plastics while producing non‐dangerous by‐products (Moharir and Kumar, 2019). These organisms could therefore find use in large‐scale procedures such as reactor systems which are commonly adopted for organic waste treatment. Despite this, very little is known about how microbial cells interact with plastics and how ‘plastisphere’ (Amaral‐Zettler *et al*., 2020) microorganisms transform these anthropogenic polymers.

Previous research has explored the taxonomic composition and spatiotemporal patterns of plastic‐colonizing microorganisms in aquatic environments using microscopy and molecular biology tools (Zettler *et al*., 2013; Amaral‐Zettler *et al*., 2015; Oberbeckmann *et al*., 2018; Coons *et al*., 2021). Results from these studies have indicated that microbes inhabiting the plastisphere reflect the local environments. These communities appear to be highly variable in taxa dominance compared with the populations in the surrounding water, with plastic‐type having a more minor influence on the community composition (Amaral‐Zettler *et al*., 2015; Coons *et al*., 2021). Importantly, plastic debris has been shown to serve as a substrate for colonization and spread of opportunistic human pathogens and bacteria related to antibiotic resistance, suggesting that these pollutants could constitute a vector for horizontal gene transfer (Zettler *et al*., 2013; Oberbeckmann *et al*., 2018).

Despite the hydrophobic nature of plastics, microbes readily colonize immersed plastics, and biofilms rapidly develop on these manufactured surfaces making them less hydrophobic and more neutrally buoyant (Lobelle and Cunliffe, 2011). On the other hand, organic ligands such as extracellular polymeric substances (EPS) produced by bacteria have also been shown to aggregate MPs in laboratory conditions and marine water from environmental samples (Summers *et al*., 2018). Moreover, the entrapment of small plastic particles in microbial‐rich organic aggregates such as marine snow (Ward and Kach, 2009; Long *et al*., 2015) has been suggested as a possible mechanism for MP export from the top layers of ocean surface waters (Zhao *et al*., 2017). As a result, since these microbial agglomerates are readily consumed by zooplankton, fish larvae and benthic‐feeding bivalves (Turner, 2015), these communities may represent an efficient vector for the trophic transfer of MPs to food chains.

While the effect of ionic strength of the aqueous environment on the aggregation of particles is well documented, and such factors are likely to have a direct or indirect impact on MP aggregation, studies that thoroughly examine the influence of MP–bacteria interactions on MPs aggregation are currently lacking. Despite the inherent complexity of these processes, it is now possible to experimentally examine the nature of MPs interactions and association with microbial aggregates. In this study we have employed carefully controlled laboratory manipulations and bioengineered platforms with inbuilt simplifications that enabled us to quantitatively analyze several aspects of the interplay between bacteria and plastic microspheres in aquatic environments. The proxy measurements generated can then be compared to other platforms and ultimately validated against environmental outcomes. The data generated could inform the creation of predictive models that can describe the dynamics and influence of bacteria on MP aggregation.

## Results and discussion

### Microplastic agglomeration by microorganisms from aquatic environments

Previous studies have described agglomeration of MPs as part of suspended biofilms in aquatic environments (Lobelle and Cunliffe, 2011; Zettler *et al*., 2013; Ikuma *et al*., 2015). To explore the process of MPs capture by microorganisms in aquatic environments, green fluorescent PS microspheres were exposed to different bacterial species (Table 1) in a laboratory‐based study. 0.5 μm diameter microspheres were chosen as model MPs since their small dimensions render inertia negligible and Brownian motion delays the precipitation which would be observed for bigger particles (Russel, 1981; Summers *et al*., 2018). These conditions permitted a more prolonged exposure to microbial cells and a more uniform distribution of the MPs in suspension.

**Table 1:** Bacterial strains used in microplastics (MPs) agglomeration studies in ASW with shaking on a roller mixer.

Bacteria species	Strain designation	Reference(s)	Aggregation time
*Cobetia marina*	ATCC 25374	Cobet *et al*. (1970), Arahal *et al*. (2002)	1 h (>0.5 h)
*Phaeobacter gallaeciensis*	CIP 105210	Ruiz‐Ponte *et al*. (1998), Martens *et al*. (2006)	3 h (>1 h)
*Pseudomonas aeruginosa*	PAO1	Holloway (1955)	2.5 h (N.a.)
*Pseudomonas* sp.	NCIMB 1084, 685	Anderson (1962)	0.66 h (>1 h)
*Pseudomonas* sp.	NCIMB 294, B‐24	MacLeod *et al*. (1954)	2 h (>0.5 h)
*Tenacibaculum discolor*	20J	Romero *et al*. (2011)	0.5 h (>4.5 h)
*Tenacibaculum maritimum*	NCIMB 2153	Wakabayashi *et al*. (1986), Suzuki *et al*. (2001)	0.83 h (>5 h)
*Vibrio campbellii*	ATCC BAA‐1116, BB120	Bassler *et al*. (1997)	1.4 h (>1 h)

The ‘Aggregation time’ column indicates times when clumping was first noticed in the microbe/MPs mixtures and in brackets the time difference when cellular aggregation was visible in vials with bacteria only. N.a. indicates that no perceptible aggregation was observed in cultures with PA only after 24 h.

**Fig. 1:**
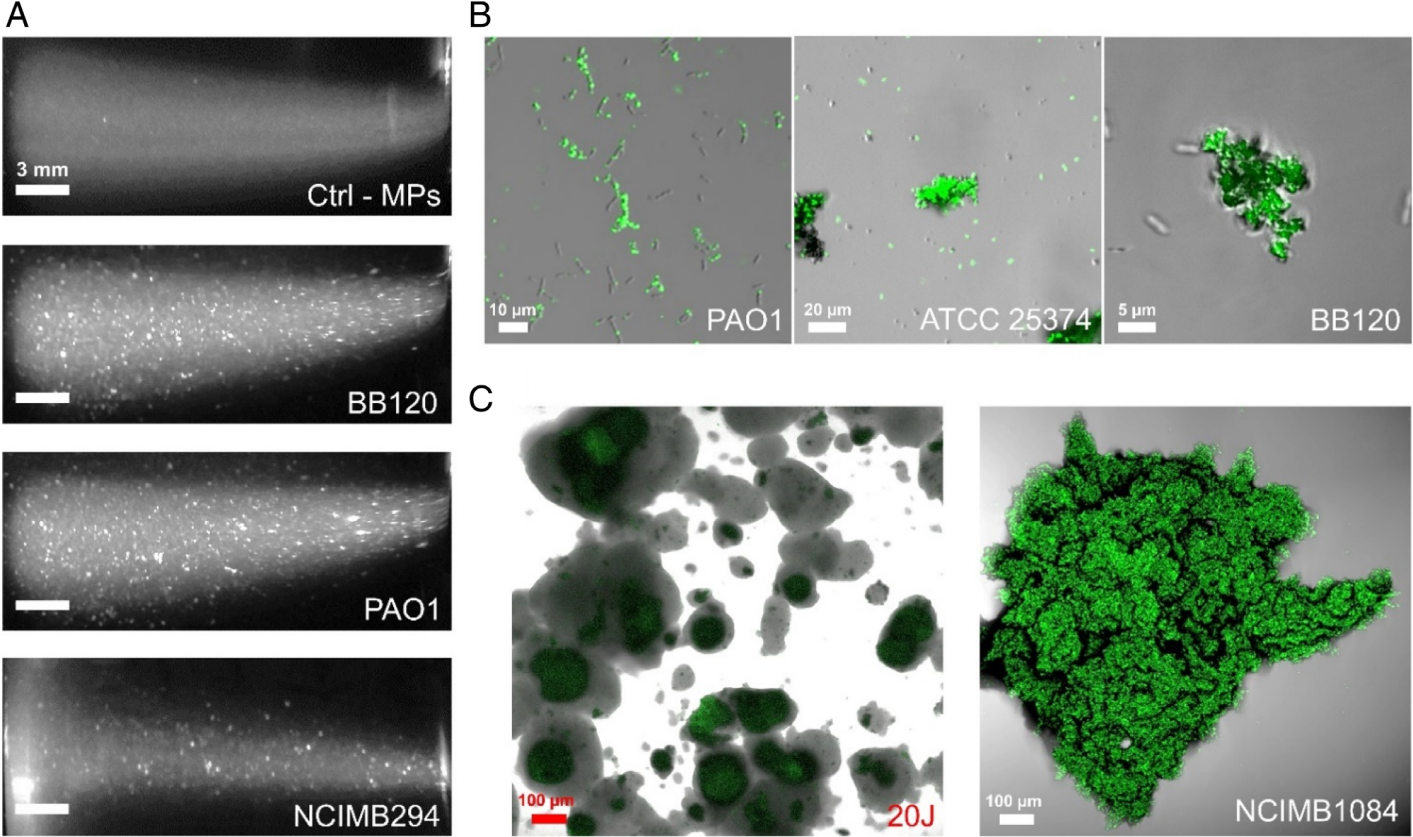
Microplastics (MPs) and bacterial cells agglomeration in artificial seawater (ASW). A. Representative images of the aggregation observed in 8 ml vials containing 10^8^ ml^−1^ MPs (0.5 μm) in ASW without (Ctrl – MPs) or with microbial cells from the indicated strains after 3 h incubation on a roller mixer. B. Confocal images of early (0.5–2 h) bacteria‐MPs agglomerates sampled from microbial and plastics mixtures in ASW. Green fluorescent signal corresponds to labelled MPs. C. Large (~0.1–1 mm) clusters of MPs (green) recovered from bacteria + MPs mixtures in ASW after 24 h incubation in rolled vials. Strains shown: *Vibrio campbellii* BB120, *Pseudomonas aeruginosa* PAO1, *Cobetia marina* ATCC 25374, *Tenacibaculum discolor* 20J, *Pseudomonas* sp. NCIMB294 and NCIMB1084.

Initially, 8 ml vials with 10 μg ml^−1^ of MPs (~1.4 × 10^8^ mL^−1^) were incubated in artificial seawater (ASW) on a roller mixer and in the presence of bacterial cells at 0.01 optical density (OD_600nm_). Representative strains from a number of species including *Cobetia marina*, *Phaeobacter gallaeciensis*, *Pseudomonas* sp., *Tenacibaculum* sp. and *Vibrio campbellii* (Table 1) well known for their ability to form biofilms in both natural and artificial environments were used (Magin *et al*., 2010; Romero *et al*., 2011; Thole *et al*., 2012; Levipan *et al*., 2019). Visual inspection showed that the rate of aggregation in ASW was enhanced in vials containing both bacteria and MPs when compared with control samples of either bacteria (0.5–5 h earlier; Table 1; Fig. 1A and B) or MPs only, which started to clump after 6–9 h incubation. This suggests that MPs enhance cellular aggregation under the conditions tested. To verify that MPs were being incorporated into the suspended aggregates, cultures with visible clumps were sampled and observed using confocal laser scanning microscopy. Results confirmed that the fluorescent MPs form a significant part of the cellular aggregates (Figure 1B and C).

### PA as a model system for testing the role of MPs in aggregate formation

Interestingly, *Pseudomonas aeruginosa* (PA) was the only bacterium species that did not show any signs of perceptible cellular aggregation in control cultures in ASW without MPs after up to 24 h incubation. Since PA cells did not aggregate by themselves under the tested conditions, the use of this species as a model system enables the effect of MPs on bacterial aggregation to be more easily assessed. Moreover, since this ubiquitously distributed opportunistic pathogen can be isolated from seawater (Kimata *et al*., 2004; Nonaka *et al*., 2010; Marathe *et al*., 2018) but also from freshwater environments (Shrivastava *et al*., 2004), interactions of this bacterium with MPs could also be assessed in salt‐free water, thereby avoiding complications due to the MPs self‐aggregation observed under high electrolyte concentration environments (Oncsik *et al*., 2015; Shams *et al*., 2020).

Initial tests of the PA cells (OD_600nm_ = 0.01, 4.8 × 10^7^ cfu ml^−1^) with 1.4 × 10^8^ MPs ml^−1^ in deionized water were carried out in rolled vials. Visual observation showed that MPs and PA cells in deionized water began to aggregate over similar time scales, but final aggregate size was slightly smaller when compared to ASW. Control cultures of just MPs or bacterial cells were also assessed under the same conditions. Importantly, no aggregation was observed in the control samples.

**Fig. 2:**
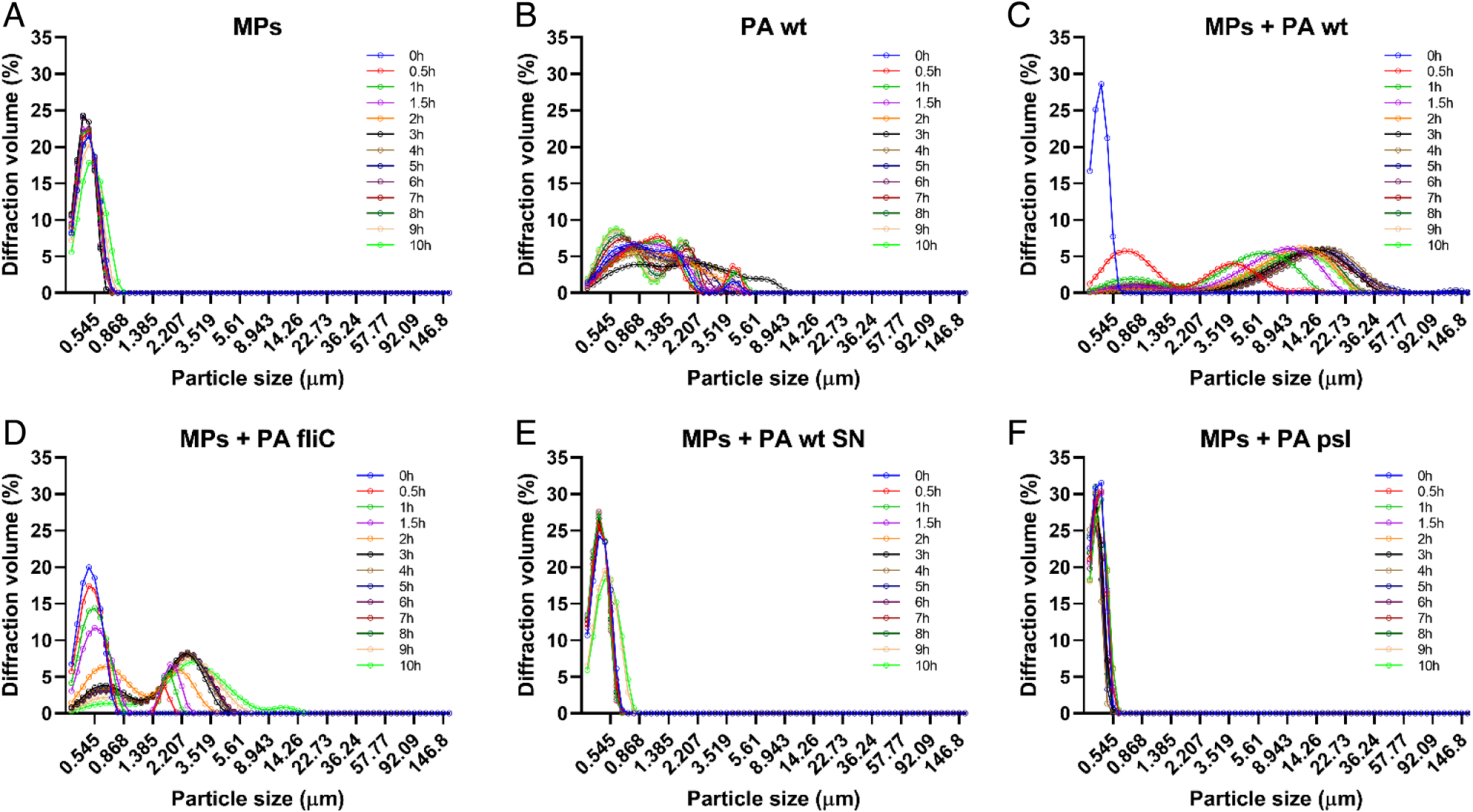
Size distribution of suspended individual particles measured using a LD analyzer over a period of 10 h. A. Geometrical dimensions of particles in distilled water supplemented with 0.5 μm MPs 1.4 × 10^8^ ml^−1^. B. Size distribution of *Pseudomonas aeruginosa* wild‐type (PA wt) cells in distilled water (4.8 × 10^7^ cfu ml^−1^). C. Changes in the size distribution of particles in a mixture of PA wt cells and MPs in distilled water over time. D. Agglomeration of MPs when exposed to the non‐flagellated *fliC* mutant strain of PA in distilled water. E. Size distribution of MPs exposed to 24 h cell‐free supernatants from agglomerated cultures of MPs and PA wt cells in distilled water. F. Size distribution of MPs exposed to PA lacking the exopolysaccharide Psl in distilled water.

Clumping of MPs and PA mixtures was also tested in vials incubated under quiescent [In later experiments (Passive flows play an important role for MPs‐bacteria aggregation section) we investigate if the static incubation used really mean absence of liquid flow in the system.] conditions, which allowed monitoring of aggregation in real‐time. Whilst slower (~5 h), PA cells still formed visible aggregates with MPs. To verify that no spontaneous aggregation of MPs occurs in freshwater, and to monitor MP agglomeration in the presence of PA cells, the size distribution of suspended individual particles/aggregates was recorded as a function of time under quiescent conditions using a laser diffraction (LD) analyzer. In agreement with previous observations, no measurable variation in particle size in control cultures of just MPs or bacterial cells occurred over a period of 10 h (Figure 2A and B). In contrast, aggregation was recorded in vials with PA and MP mixtures. Rapid agglomeration occurred (<0.5 h) and the culture reached a maximum average particle size after ~5 h of incubation (Fig. 2C).

### Removal of MPs from suspension

**Fig. 3:**
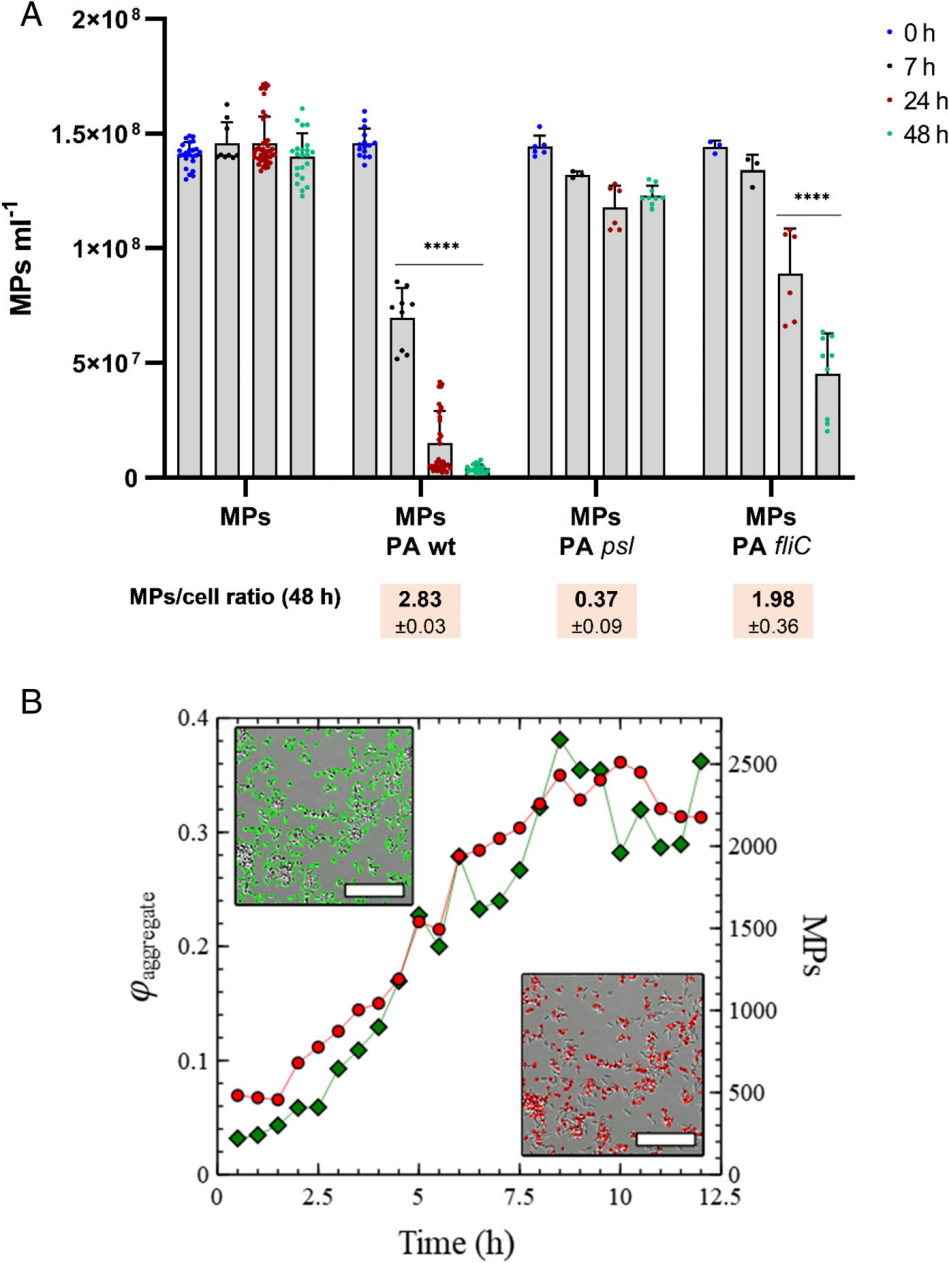
Sedimentation of MPs aggregates exposed to PA cells over time. A. Number of MPs remaining in solution after different time exposures to parental and mutant strains of PA cells incubated under quiescent conditions. The average MP per cell ratios ± SD after 48 h incubation for each strain is shown. Statistical differences between group means were determined by two‐way ANOVA tests (*p < 0.05; **p < 0.01; ***p < 0.001; ****p < 0.0001). B. Progressive increase in the number of MP‐PA sedimenting clusters (green diamonds) and the number of sedimenting MPs (red circles) over a period of 12 h. Insets include representative 6.5 h DIC microscopy images from the surface of a glass‐bottom Petri dish. Image processing was used to analyze the obtained images to quantify the total fraction of the surface covered by aggregates (green) and the total number of MPs (red). The scale bar shown in each inset represents 10 μm.

Sedimentation of MPs in aquatic environments has important implications for their mobility and dispersal (Zhao *et al*., 2017). If interactions with microorganisms alter the sedimentation characteristics of MPs this has the potential to impact their transit and prevalence in local ecosystems (Lobelle and Cunliffe, 2011; Long *et al*., 2015; Kooi *et al*., 2017). LD data indicated that the number of MPs in suspension remained relatively constant in the absence of bacteria, implying stability to sedimentation on the timescales studied. However, in suspensions containing PA cells under quiescent conditions, MPs were found to undergo sedimentation from suspension (Fig. 3A).

Samples containing mixtures of MPs and PA bacteria were also incubated in a glass‐bottom Petri dish over a 12 h period. At the same time, the bottom surface of the dish was repeatedly imaged using time‐lapse differential interference contrast (DIC) microscopy. Consistent with LD data, image analysis allowed us to quantify the progressive increase in the number of surface deposited clusters and also the number of MPs present from early incubation time points (Fig. 3B). Plots of the number of MPs and the increase in area fraction covered by aggregates exhibit a similar dependence on incubation time. This suggests that the sedimentation of MPs occurs mainly through association with bacteria. This is further supported by the observation that MPs were found to be almost exclusively associated with PA aggregates and are rarely observed absorbed to the surface as isolated MPs (see insets Fig. 3B).

To understand the aggregation and subsequent sedimentation process we performed a series of experiments to:

- Assess how the MPs and PA in suspension interact and come to adhere to one another.
- Elucidate the factors responsible for aggregate growth.

### The role of EPS in MP‐bacteria aggregation

A recent study (Summers *et al*., 2018) reported EPS from marine water, and probably produced by microorganisms (Decho and Gutierrez, 2017), as a significant component of nano‐ and microplastic agglomerates and therefore an essential agent in understanding MP aggregation within aquatic environments. It was also noted, by visual inspection, that there was an increase in medium viscosity when supplemented with purified EPS, resulting in the hypothesis that this might affect aggregate size and stability.

To understand the possible role of EPS in MP aggregation by PA we tested whether our system exhibited significant viscosity changes. To assess this, supernatants were obtained from 24 h suspensions containing PA cells and MPs in deionized water under rolling and non‐rolling conditions. Fresh MPs were then suspended in these supernatants and the motion of the particles was tracked in glass‐bottom dishes using DIC microscopy. Standard single‐particle microrheology (Cicuta and Donald, 2007) was then used to measure the viscosity of the medium. Results were then compared to MPs motion in fresh deionized water under the same conditions. The viscosity measurements indicated that released EPS substances resulted in no significant change in liquid viscosity (Supplementary Fig. 1). This suggests that EPS + induced changes to medium viscosity plays little or no role in MPs aggregation under the conditions tested in this study.

Whilst EPS substances do not alter the medium viscosity it is possible that they still play a role in reducing colloidal stability, thus promoting aggregation. We tested this in two complementary ways: First, media from aggregated 24 h cultures of MPs and PA cells were recovered and filter‐sterilized. The media was then used to suspend fresh MPs without PA cells. LD data showed no aggregation of MPs exposed to spent supernatants of PA for 10 h (Fig. 2E). Second, since the exopolysaccharide Psl is one of the main EPS substances produced by the PAO1 strain of PA used in this study (Colvin *et al*., 2012), agglomeration of MPs was tested under increasing concentrations of purified Psl (1–100 μg ml^−1^) in water. Moreover, as Psl is produced as a low‐ and high‐molecular‐weight polysaccharide both fractions were used in the assays. Fluorescence readings of the quiescent suspensions incubated for 24 h showed that Psl supplementation did not yield a reduction in the number of MPs in solution compared to the negative control (Supplementary Fig. 2A). These results suggest that, in the case of PA, MPs clumping is not triggered by a factor released into solution by the bacterial cells or that the EPS substance(s) released are not by themselves sufficient to produce MP aggregation. Interestingly, these results agree with previously published data showing that a purified glycoprotein polymer produced by the marine bacterium *Halomonas* sp. TGOS‐10 did not produce an increase in the number of plastic agglomerates formed in marine water compared to controls without EPS supplementation (Summers *et al*., 2018).

### Cell‐associated Psl and MP aggregation

The exopolysaccharide Psl is made of pentasaccharide subunits that contain mannose, rhamnose and glucose in the ratio 3:1:1 (Ma *et al*., 2007). Furthermore, Psl can be secreted or found cell‐associated and constitutes a bonding agent produced in response to cell contact with surfaces (Byrd *et al*., 2009; Armbruster *et al*., 2019). Therefore, we hypothesised that although EPS substances in suspension did not promote MP clumping, cell‐associated Psl may still be necessary for PA to adhere to MPs.

To test this possibility, agglomeration of MPs by a *psl* operon promoter mutant of PA (Ma *et al*., 2006) was monitored employing LD. Using this strain, which lacks the ability to produce EPS Psl, we did not detect any aggregate formation over an incubation period of 10 h (Fig. 2F). Fluorescence readings were also used to estimate the amount of MPs remaining, in the same types of suspensions, after different times. We detected no significant reduction in the number of MPs in suspension contrary to the wild‐type strain (Fig. 3A).

**Fig. 4:**
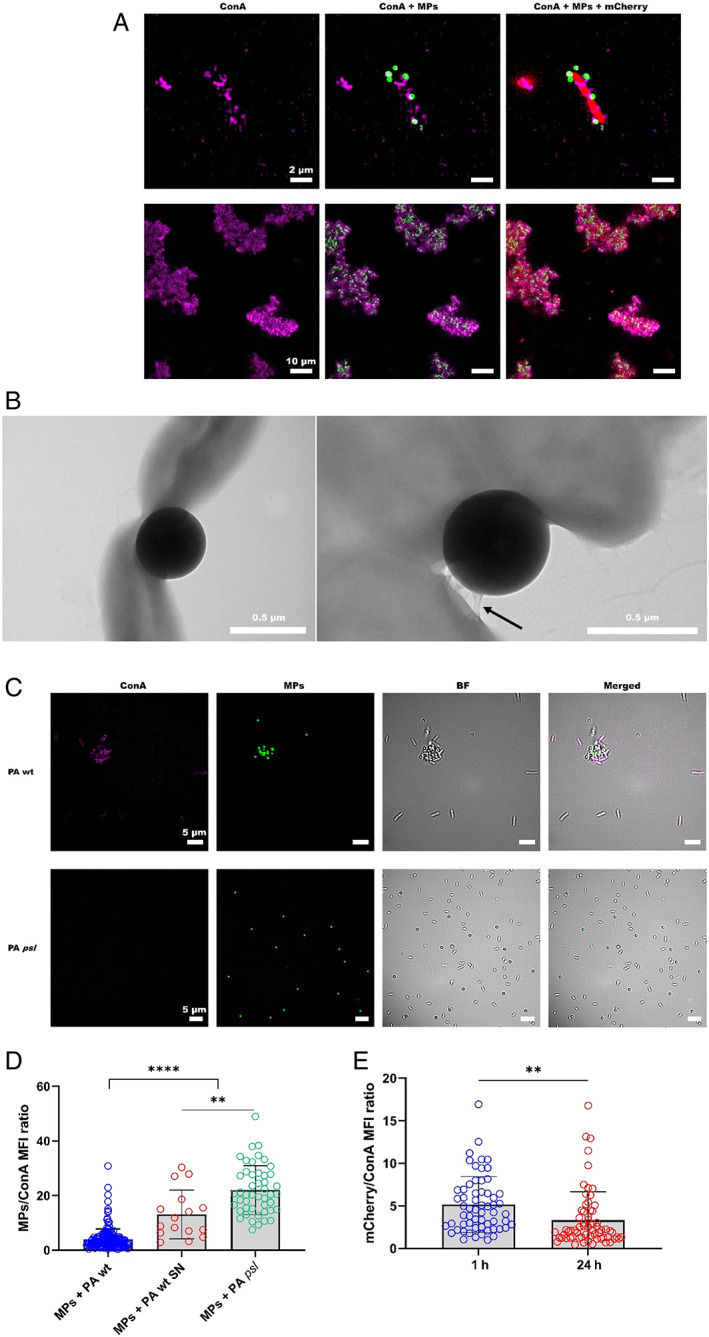
PA Psl exopolysaccharide and MPs‐cells aggregation. A. High‐resolution images of MPs (green) attached to individual cells (top row) and cellular aggregates (bottom row) of *Pseudomonas aeruginosa* (PA) expressing the fluorescent protein mcherry (red). Cell‐associated exopolysaccharide labelled with concanavalin A (ConA) lectin is shown as purple. B. ESEM images of MPs attached to PA cells showing the MPs partially coated with exopolymeric substances (black arrow). C. Fluorescent and bright‐field (BF) images showing absence of agglomeration of MPs (green) exposed to PA lacking the ConA‐stained (purple) exopolysaccharide Psl (PA *Δpsl*) compared to the wild‐type strain (PA WT). D. Quantification of mean fluorescence intensity (MFI) of ConA associated to MPs exposed to PA wild‐type cells (PA wt), PA supernatants (SN) or the PA strain unable to produce the exopolysaccharide Psl (PA *psl*) for 24 h. E. Quantification of cell‐associated ConA mean fluorescence intensity (MFI) after incubation of PA with MPs for 1 and 24 h. Data shown are mean ± SD. Statistics are two‐tailed Student's *t*‐test (*p < 0.05; **p < 0.01; ***p < 0.001; ****p < 0.0001).

PA‐MPs aggregates were also fluorescently labelled with concanavalin A (ConA) lectin, which associates with exopolysaccharides containing α‐d‐mannosyl and α‐d‐glucosyl groups. High‐resolution microscopy and ESEM images of individual microbial cells and aggregates showed layers of exopolysaccharide on the cells surface and assemblies of exopolysaccharide appeared to bond the MPs with PA cells (Figure 4A and B). These structures were absent in the *psl* mutant strain, suggesting that EPS is necessary for cells to attach to MPs (Fig. 4C). Moreover, higher quantities of exopolysaccharide were found associated with MPs exposed to PA cells compared to those interacting with the *psl* mutant or wild‐type supernatants (Fig. 4D). To evaluate whether MPs stimulate Psl production, exopolysaccharide production was estimated as a ratio of ConA‐stained Psl to a constitutively expressed *mcherry* reporter in PA (Popat *et al*., 2012). A significant increase in Psl production by PA cells was recorded after 24 h incubation with MPs (Fig. 4E). These findings suggest that interaction with MPs stimulates EPS production by the microbial cells and, consequently, more captured MPs could end up entrapped by these materials and the cells producing them.

To assess whether microparticle capture by cell‐associated Psl could enable aggregation of particles with different chemical properties and to approximate a non‐synthetic surface present in aquatic environments such as sand, aggregation of 0.5 μm silica microspheres by PA was also tested for 24 h under rolling conditions in water. Despite their non‐hydrophobic nature, SiO_2_ microparticles were aggregated by PA wild‐type strain and, similarly to PS MPs, exposure to the *psl* mutant strain of PA produced no agglomeration of silica (Supplementary Fig. 3). Therefore, it seems that adhesion by cell‐associated Psl could be a common process for aggregating surfaces with different chemical compositions in PA. However, it remains to be studied if this is a universal mechanism for capturing other plastic microparticles by PA and if the cell‐associated expression of EPS substances is also necessary to promote adhesion to MPs by other EPS‐producing bacteria from aquatic environments.

It is well known that after bacteria sense their attachment to a surface, they initiate the switch to a sessile lifestyle by altering their physiological status (Petrova and Sauer, 2012; Persat *et al*., 2015). This process is governed by cyclic diguanylate (c‐di‐GMP) levels controlling phenotypic changes related to the transition between motility and sessility, with high levels promoting biofilm formation traits such as EPS release in bacteria (Romling *et al*., 2013; Valentini and Filloux, 2016). We therefore questioned whether PA cells sense attachment to the MP particles, as suggested by the significant quantities of Psl produced in PA‐MPs agglomerates. To assess this *P*. *aeruginosa* PAO1 carrying the transcriptional fusion P*cdrA*‐*gfp*(ASV)^S^, which reports intracellular c‐di‐GMP levels in the cells, was exposed to MPs in water. As shown in Supplementary Fig. 2B, PA cells displayed high levels of *cdrA* expression after 10 h of incubation with MPs, well above that of cells incubated for 2 h or from planktonic cultures without MPs, consistent with elevated c‐di‐GMP levels. This result provides a mechanistic understanding of how cells sensing MPs adhesion may enhance EPS release by PA.

### Is aggregate growth passive or active?

Given that PA cells are flagellated and therefore motile, it is an interesting question whether aggregate growth is governed by passive processes such as MP diffusion, and convective flows within the sample or relies on the motility of PA. To assess the importance of bacterium motility, samples were prepared containing MPs and cells from a *fliC* mutant of PA lacking flagella appendages (Romero *et al*., 2022). Aggregate formation was then monitored using LD. Figure 2D shows that in common with the PA parental strain, the non‐motile *fliC* strain could also cluster MPs; however, the aggregation rate was reduced. To study the number of particles precipitated by the *fliC* mutant, samples from cultures incubated under quiescent conditions were collected. The number of MPs remaining in the solution was determined by quantifying the fluorescence signal of the samples. In agreement with LD measurements, fluorescence readings indicated that the non‐motile strain could precipitate MPs slower than the parental strain in non‐shaken conditions (Fig. 3A). These findings suggest that cell motility is not strictly necessary for MPs capture by PA cells in a liquid medium. However, the increase in precipitation observed for the wild‐type strain suggests cell motility increases the probability of collision events leading to MP capture and thereby enhancing aggregation rates.

### Passive flows play an important role for MPs‐bacteria aggregation

Despite our results indicating that motility alters the dynamics of aggregation, it is clear that passive processes (e.g. diffusion) must also be important. In our earlier results we found differences in the rate of aggregation of samples incubated under quiescent or rolling conditions. Although dishes with MPs were maintained in stationary settings during the tracking experiments mentioned above, streams of MPs were observed to slowly move in small convection currents. These were not remitting even after extended incubations (>24 h). This observation, together with the fact that microbial motility was not required for MPs capture by PA (Fig. 2D), suggested that residual flows present in still vials could influence the formation of agglomerates.

In an attempt to mitigate liquid currents, closed chambers were built and assessed to reduce residual flows. We found that MPs and PA mixtures incubated under quiescent conditions in chambers of <1 mm in height and without air–liquid interphases had no observable streams of particles and therefore were selected to evaluate MPs agglomeration. Without liquid currents, no aggregates of MPs and bacterial cells were observed even after 24 h incubation (Supplementary Fig. 4). This result suggests that liquid flows could promote aggregate growth by preventing sedimentation, thereby keeping the early MPs‐PA clusters formed exposed to further bacterial cells and MPs attachment in suspension. Once the aggregates reach a critical size (~10–20 μm, Fig. 2C), the diffusion present in our setup could not keep the aggregates in suspension and they precipitate out of solution. The absence of flows in closed chambers would make initial clusters precipitate earlier and therefore no significant agglomeration is observed (Supplementary Fig. 4).

Importantly, these results could potentially explain the observed discrepancy between predicted and traceable amounts (one to three orders of magnitude lower) of MPs in the top layers of aquatic environments (Jambeck *et al*., 2015; Zhao *et al*., 2017). They provide a mechanistic picture in which water currents in aquatic environments enhance MP clustering and capture by microorganisms, thereby accelerating aggregate growth and settling of plastic. This could constitute a key process for exporting this buoyant debris from surface waters.

### The influence of aggregate dynamics on aggregation

Given that PA cells can sense attachment to the plastic particles, as indicated by MPs stimulated c‐di‐GMP production and the significant quantities of Psl produced in PA‐MPs agglomerates, this could translate into a decrease in motility. Agglomerates of bacteria and MPs would therefore be largely passive objects diffusing in the medium. Growth would mainly occur via individual bacteria/MPs periodically joining the large aggregates rather than the motion of aggregates themselves contributing to the process.

**Fig. 5:**
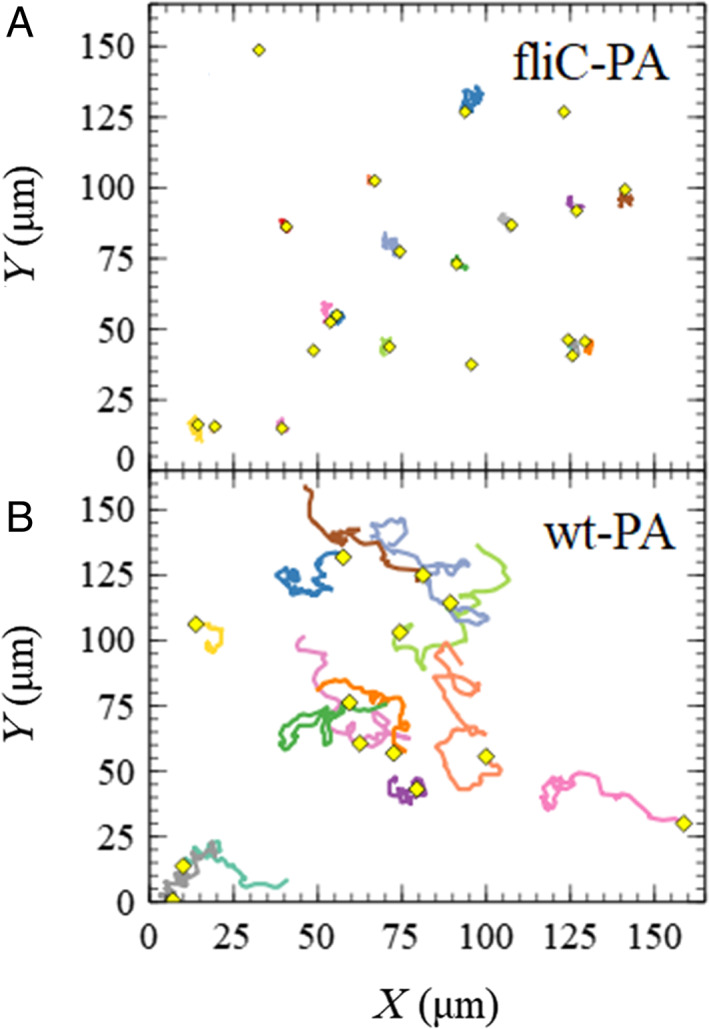
Trajectories of preformed 24 h aggregates of MPs + PA cells from the wild‐type (A) and the non‐motile *fliC* mutant strain of PAO1 (B). The average diameters for the tracked aggregates were approximately 15 ± 7 μm for the wild‐type strain and 12 ± 3 μm for the *fliC* mutant. The trajectory of each aggregate is shown for 25 s. The initial position of each aggregate is indicated by the yellow diamond.

Although a speed reduction was noticed, the motion of individual PA cells was not severely hampered by early attachment to a small number of MPs as they were observed to swim and carry the MPs attached using DIC optical microscopy (Supplementary Videos). Bigger aggregates of bacteria and MPs were also prepared by incubation for 24 h using the motile PA wild‐type strain and the other using the non‐motile *fliC* strain. Aliquots were transferred to a sample containing pure water and imaged using a confocal microscope at frame rates of 4.13 fps for periods ~25 s. Particle tracking was then used to assess the motility of the aggregates. Figure 5 shows the trajectories of several different aggregates of both types. The aggregates containing the motile strain (PA‐MPs) were on average slightly larger (approximate diameter 15 ± 7 μm) than those formed from the non‐motile cells *fliC*‐MPs (12 ± 3 μm of diameter). Diffusion of a spherical particle in suspension decreases with increasing diameter. Despite this, the PA‐MPs showed motion an order of magnitude larger than the corresponding *fliC*‐MPs. This demonstrates that bacteria agglomerates still have significantly enhanced motility when compared with passive aggregates of the same size and therefore have not entered a completely sessile state. The motion of aggregates containing motile cells could therefore still contribute to their growth. This represents an important additional aspect if the aggregation and transport of MP–bacteria aggregates are to be accurately modelled, since the aggregates cannot be described as passively diffusing but remain active.

## Conclusions

In this study we have shown that even under idealized conditions, that maximize colloidal stability of both MPs and bacteria (PA) individually, suspensions containing both resulted in rapid aggregation. Focussing on the interaction between PA and MPs we showed that, whilst EPS does not affect aggregation by altering colloidal stability, cell‐associated expression of EPS (Psl) is necessary to promote adhesion to MPs and initiate biofilm formation. The growth of aggregates was found to be enhanced by bacterial motility but could also occur even in non‐swimming bacterial strains provided sufficient flows existed within the liquid. Perhaps the most surprising result is that the motile PA strains do not appear to enter a sessile state upon attachment to MPs despite the observed production of EPS and initial aggregate formation. Whilst our experiments were mostly performed in pure water it seems likely that our results, at least qualitatively, identify the important mechanisms behind aggregation more generally, at least for the MPs and bacterial strains considered. It is hoped that these mechanistic insights will inform future modelling of the aggregation and transport of MPs in the presence of bacteria and reveal new approaches for managing plastic pollution.

## Materials and methods

### Bacterial strains and culture conditions

The following strains were used to explore the process of MPs agglomeration by bacteria: *Cobetia marina* ATCC 25374, *Phaeobacter gallaeciensis* CIP 105210, *Pseudomonas aeruginosa* PAO1, *Pseudomonas* sp. NCIMB 1084, *Pseudomonas* sp. NCIMB 294, *Tenacibaculum discolor* 20J, *Tenacibaculum maritimum* NCIMB 2153 and *Vibrio campbellii* BB120. Bacteria were routinely cultivated in marine broth (PanReac) or lysogeny broth in the case of *P*. *aeruginosa* PAO1 (PA).

To simulate marine water, ASW was prepared by dissolving 30 g NaCl; 0.8 g KCl; 6.6 g MgSO_4_; 0.5 g NaHCO_3_ and 1.3 g CaCl in 1 L of water (Tesfaye *et al*., 2018). PS fluorescent microspheres (1.4 × 10^8^ particles ml^−1^, Ø 0.5 μm – Fluoro‐Max™ Green fluorescent, Thermo Scientific) were exposed to bacteria with OD at 600 nm of 0.01 in ASW or deionized water in 8 ml scintillation vials. Bacteria and MPs were incubated at RT under quiescent or dynamic conditions (33 rpm) using a roller and rocker mixer (SRT6, Stuart) in a process similar to a laboratory‐made artificial marine snow method described before (Shanks and Edmondson, 1989). Vials were visually inspected using a white light transilluminator to monitor for signs of MPs agglomerate formation. To model a non‐synthetic surface present in aquatic environments such as sand, silica‐based (SiO_2_) microparticles (1.4 × 10^8^ particles ml^−1^, Ø 0.5 μm – Merck) were exposed to PA cells at OD 0.01 in deionized water in 8 ml scintillation vials for 24 h under rolling conditions.

To reduce convection in the water due to evaporation, ~1 mm height closed chambers were built by mounting two glass coverslips (24 × 50 mm) on double‐sided adhesive. Chambers were filled with PA (OD_600nm_ 0.01 = 4.8 × 10^7^ cfu ml^−1^) and MPs (1.4 × 10^8^ particles ml^−1^) mixtures and sealed with silicone and halogen‐free high‐vacuum grease (Apiezon M) to avoid air access and water evaporation.

### Laser diffraction measurements

The size of the agglomerates formed in PA and MPs mixtures was analyzed with a LD particle size analyzer (Beckman‐Coulter LS230). To study the influence of bacterial motility and EPS production on MPs capture, a *fliC* mutant lacking flagella appendages (Romero *et al*., 2022) and a *psl* operon promoter mutant of PA (Ma *et al*., 2007) were used respectively. Wild‐type and mutant PA strains (4.8 × 10^7^ cfu ml^−1^) were exposed to 1.4 × 10^8^ MPs ml^−1^ in a 12 ml cell module, and agglomerate formation was analyzed in real‐time over 10 h of incubation at RT and without sample stirring. To assess whether EPS substances released into the medium could trigger MPs aggregation, MPs were exposed to filter‐sterilized (0.22 μm) samples from clumped PA + MPs mixtures taken after 24 h incubation in deionized water.

### Microplastics agglomeration imaging and data acquisition

*Pseudomonas aeruginosa* PAO1 carrying the constitutively expressed *mcherry* gene on the plasmid pMMR (Popat *et al*., 2012) was used for imaging PA and MPs/silica clusters. Concanavalin A – Alexa Fluor™ 647 Conjugate (ConA, Invitrogen) dye was employed to stain the exopolysaccharide component of sampled aggregates. Syto9 (Invitrogen) nucleic acid dye was used to stain *psl* mutant cells exposed to silica microparticles. For high‐resolution imaging of fluorescent bacteria and MPs clusters an Elyra PS1 Super Resolution Microscope (Carl Zeiss) with C‐Apochromat 63×/1.2 W Korr M27 objective was used in structured illumination (SIM) and confocal modes. For SIM multiple fluorescent channels were configured and an additional transmitted bright field channel was used. 488 nm (1%), 561 nm (10%) and 642 nm (20%) lasers were employed to image fluorescent MPs, mCherry and Psl exopolysaccharide respectively. SIM gratings were set to 34, 42 and 51 μm and exposures to 20, 30 and 100 ms respectively. For detection, bandpass BP 495–550 + LP 750, BP 570–620 + LP 750 and long pass LP 655 filters were used. In confocal mode, 488 nm (0.5%), 561 nm (8%) and 633 nm (20%) lasers were used. A multichannel dichroic was selected 488/561/633 and detection was set as follows: green: 499–561, red (mCherry): 570–641, far red (ConA): 679–735 nm range. Detector gains were set to avoid overexposures, and small pinhole setting (24 μm) was used to obtain 0.2 μm optical slices. Zstacks were recorded, in a volume of 2.2 μm, with 0.2 μm step size. Images were visualized and exported using Zen Black 2012 (Carl Zeiss) software.

Bacteria and MPs agglomerates were also analyzed using ESEM, enabling the semi‐hydrated samples to be imaged without a conductive coating to gain insight into their native state. Clusters were fixed overnight in 2.5% EM grade glutaraldehyde buffered with 0.1 M sodium cacodylate at 4°C. Following fixation, samples were washed (3 × 5 min) in 0.1 M sodium cacodylate buffer, adhered to sample holders using die‐cut carbon conductive adhesive discs (SPI Supplies/Structure Probe) and imaged on a Quanta 200FEG SEM microscope (FEI Company). Chamber parameters were equilibrated to <−10°C and 238 Pa at 5–15 kV to progressively sublimate sample water content.

For particle tracking an inverted TE2000 microscope (Nikon) equipped with a Hamamatsu Orca Flash 4.0v2 sCMOS camera was used. DIC imaging was carried out using a single channel white MonoLED (Cairns) light source and a 40× objective (Nikon, CFI Plan Fluor 40×/1.3). Experiments were conducted using sterile glass‐bottom Petri dishes (35 × 20 × 10.5 mm – VWR) inoculated with 4 ml of MPs only or MPs + PA mixtures and incubated at RT for up to 12 h. 1000 frame videos (32 ms distance between frames) were taken every 30 min either at the bottom or 100 μm above the dish surface. To measure cell‐induced clusters motility, samples including preformed 24 h aggregates of MPs exposed to PAO1 wild‐type and non‐motile *fliC* strains were collected from stationary 8 ml vials, deposited in glass‐bottom dishes and filmed with a Carl Zeiss LSM700 confocal microscope using an EC Plan‐Neofluar 10×/0.30 M27 objective at 4.13 fps. Aggregate trajectories were tracked for 25 s. Particle tracking was performed with the ParticleTracker open source software (Smith and Downs, 2021). The aggregates were detected using a contour finding method on thresholded images.

To assess the number of plastics captured by PA cells, 0.2 ml samples from MPs and bacteria mixtures in distilled water were collected after 0, 7, 24 and 48 h incubation at RT from the top layers of 8 ml vials and the green fluorescence emitted by MPs in suspension was quantified using a 96‐well plate TECAN Genios Pro multifunction microplate reader. A standard curve relating the number of MPs and fluorescence output was set and used to estimate the quantity of MPs remaining in suspension.

To semi‐quantify c‐di‐GMP levels, the fluorescence activity of PAO1 cells carrying the plasmid P*cdrA*‐*gfp*(ASV)^S^ (Rybtke *et al*., 2012) was measured in confocal images from samples taken after 2 and 10 h incubation with or without undyed 1 μm PS particles (Sigma‐Aldrich) at concentration 1.4 × 10^8^ MPs ml^−1^.

### Data analysis

Fluorescence data from microscopy images were obtained using the open‐source software Fiji‐ImageJ v2.1.0/1.53c (Schindelin *et al*., 2012). Unpaired *t*‐tests comparisons and two‐way ANOVA tests were applied to determine whether MPs agglomeration differed significantly between incubation conditions (*p* < 0.05) when compared with the variations within the replicates using Prism 8.0 (GraphPad Software).

## Supporting information


**Supplementary Fig. 1.** Viscosity measurements based on diffusion of MPs in deionized water (blue) or PA supernatants (PA wt SN) obtained from cultures exposed to MPs for 24 h in deionized water under rolling (green) and non‐rolling (red) conditions.
**Supplementary Fig. 2.** A) Number of MPs remaining in solution after 24 h exposure to PA cells or different concentrations of low and high molecular weight fractions (LMW/HMW) of purified Psl EPS under quiescent conditions. B) Reported values are mean fluorescence intensities measured from confocal image areas occupied by PA cells carrying the transcriptional fusion P*cdrA*‐*gfp* incubated for 2 and 10 h under rolling conditions in water −/+ MPs. Statistical differences between group means were determined by two‐way ANOVA tests (*p < 0.05; **p < 0.01; ***p < 0.001; ****p < 0.0001).
**Supplementary Fig. 3.** Confocal microscopy images showing absence of aggregation of silica microparticles (white spheres) in the presence of syto9‐labelled Psl mutant cells of PA (PA *psl*, red) compared to the wild‐type strain (PA wt) expressing the fluorescent protein mcherry (red). Scale bar 5 μm.
**Supplementary Fig. 4.** Representative differential interference contrast (DIC) microscopy images showing the lack of MPs + PA cells aggregation after 24 h incubation under truly static conditions in <1 mm closed glass chambers (no flow) compared to mixtures incubated in glass‐bottom dishes with medium currents due to convection (flow). Images were taken at 0 and 100 μm high from the centre of containers' bottom glass. Scale bar 10 μm.

**Appendix** **S1**: Supporting Information.
