# Supplementary material for: Fluid dynamics and cell‐bound Psl polysaccharide allows microplastic capture, aggregation and subsequent sedimentation by Pseudomonas aeruginosa in water
Source: Environ Microbiol. 2022 Feb 2;24(3):1560–72. doi: 10.1111/1462-2920.15916 (PMC9305584; doi:10.1111/1462-2920.15916)
Supplement: Supplementary file 1 — Supplementary Fig. 1. Viscosity measurements based on diffusion of MPs in deionized water (blue) or PA supernatants (PA wt SN) obtained from cultures exposed to MPs for 24 h in deionized water under rolling (green) and non‐rolling (red) conditions. Supplementary Fig. 2. A) Number of MPs remaining in solution after 24 h exposure to PA cells or different concentrations of low and high molecular weight fractions (LMW/HMW) of purified Psl EPS under quiescent conditions. B) Reported values are mean fluorescence intensities measured from confocal image areas occupied by PA cells carrying the transcriptional fusion PcdrA‐gfp incubated for 2 and 10 h under rolling conditions in water −/+ MPs. Statistical differences between group means were determined by two‐way ANOVA tests (*p < 0.05; **p < 0.01; ***p < 0.001; ****p < 0.0001). Supplementary Fig. 3. Confocal microscopy images showing absence of aggregation of silica microparticles (white spheres) in the presence of syto9‐labelled Psl mutant cells of PA (PA psl, red) compared to the wild‐type strain (PA wt) expressing the fluorescent protein mcherry (red). Scale bar 5 μm. Supplementary Fig. 4. Representative differential interference contrast (DIC) microscopy images showing the lack of MPs + PA cells aggregation after 24 h incubation under truly static conditions in <1 mm closed glass chambers (no flow) compared to mixtures incubated in glass‐bottom dishes with medium currents due to convection (flow). Images were taken at 0 and 100 μm high from the centre of containers' bottom glass. Scale bar 10 μm. [file EMI-24-1560-s001.pdf]

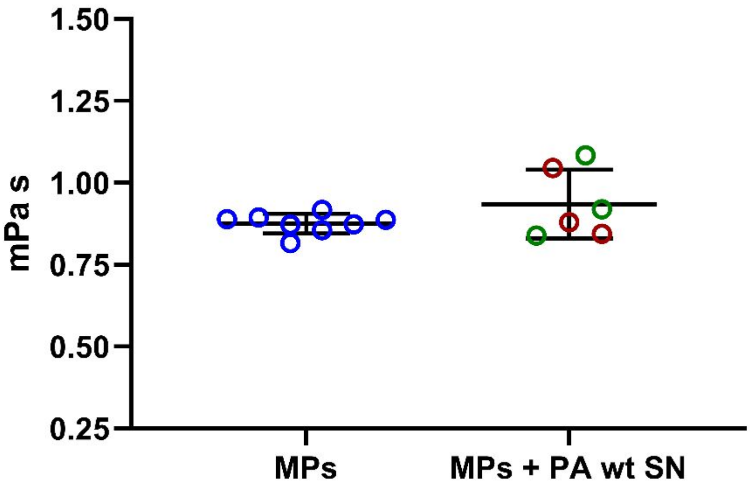

Supplementary figure 1. Viscosity measurements based on diffusion of MPs in deionised water (blue) or PA supernatants (PA wt SN) obtained from cultures exposed to MPs for 24h in deionised water under rolling (green) and non-rolling (red) conditions.

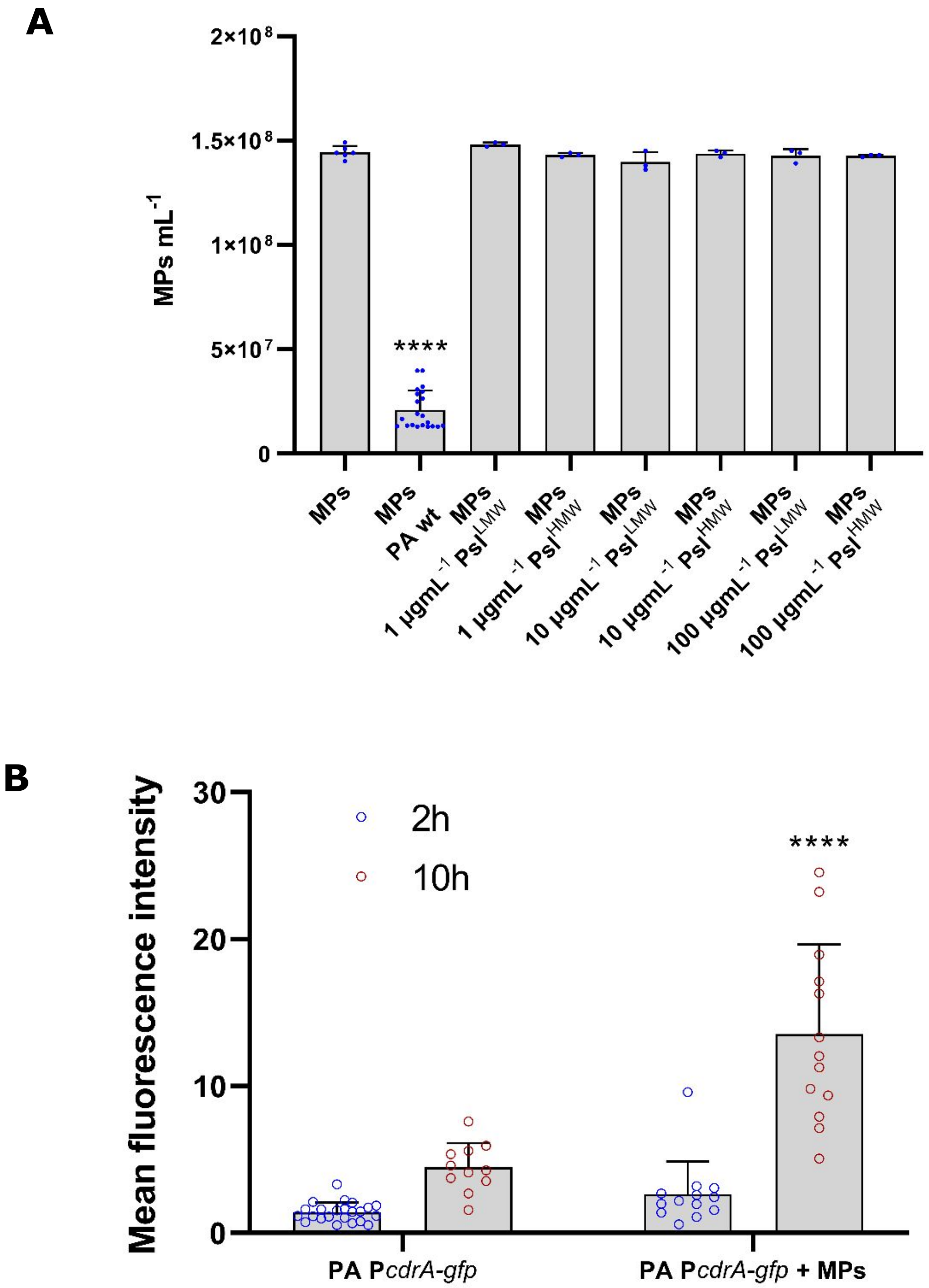

Supplementary figure 2. A) Number of MPs remaining in solution after 24h exposure to PA cells or different concetrations of low and high molecular weight fractions (LMW/HMW) of purified Psl EPS under quiescent conditions. B) Reported values are mean fluorescence intensities measured from confocal image areas ocupped by PA cells carrying the transcriptional fusion P*cdrA-gfp* incubated for 2 and 10h under rolling contions in water -/+ MPs. Statistical differences between group means were determined by two-way ANOVA tests (* p<0.05; ** p<0.01; *** p<0.001; **** p<0.0001).

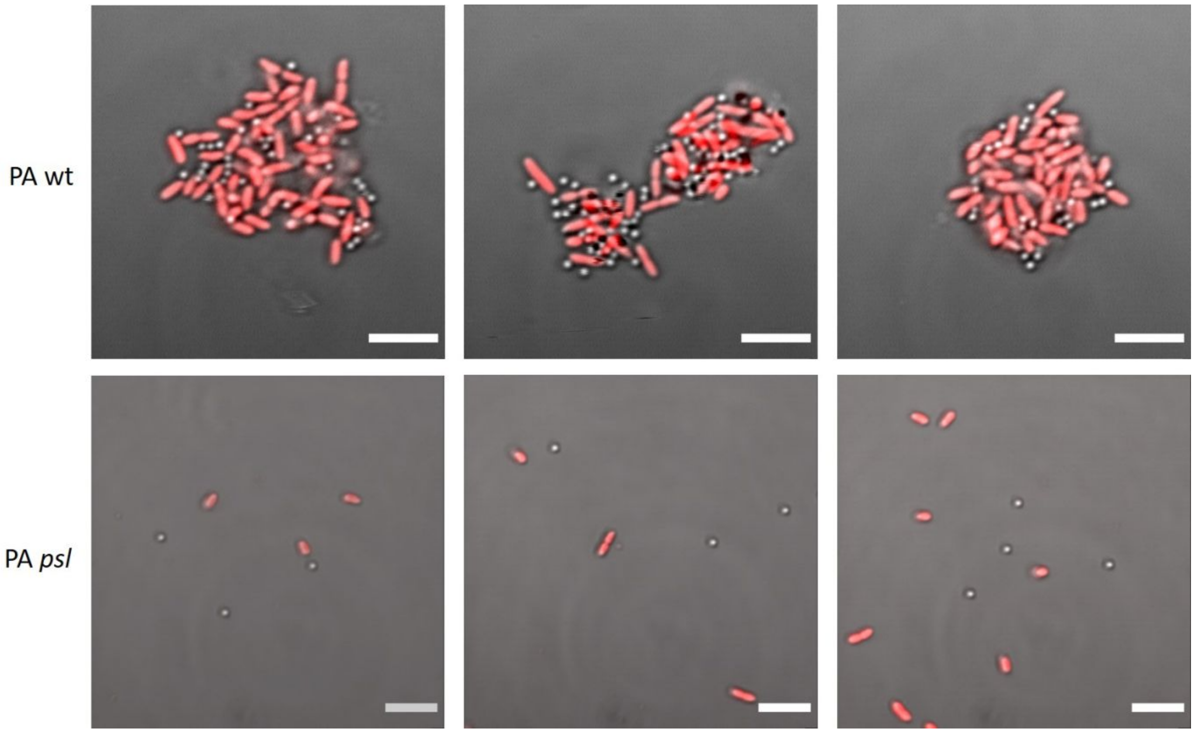

Supplementary figure 3. Confocal microscopy images showing absence of aggregation of silica microparticles (white spheres) in the presence of syto9-labelled Psl mutant cells of PA (PA *psl*, red) compared to the wild-type strain (PA wt) expressing the fluorescent protein mcherry (red). Scale bar 5 μm.

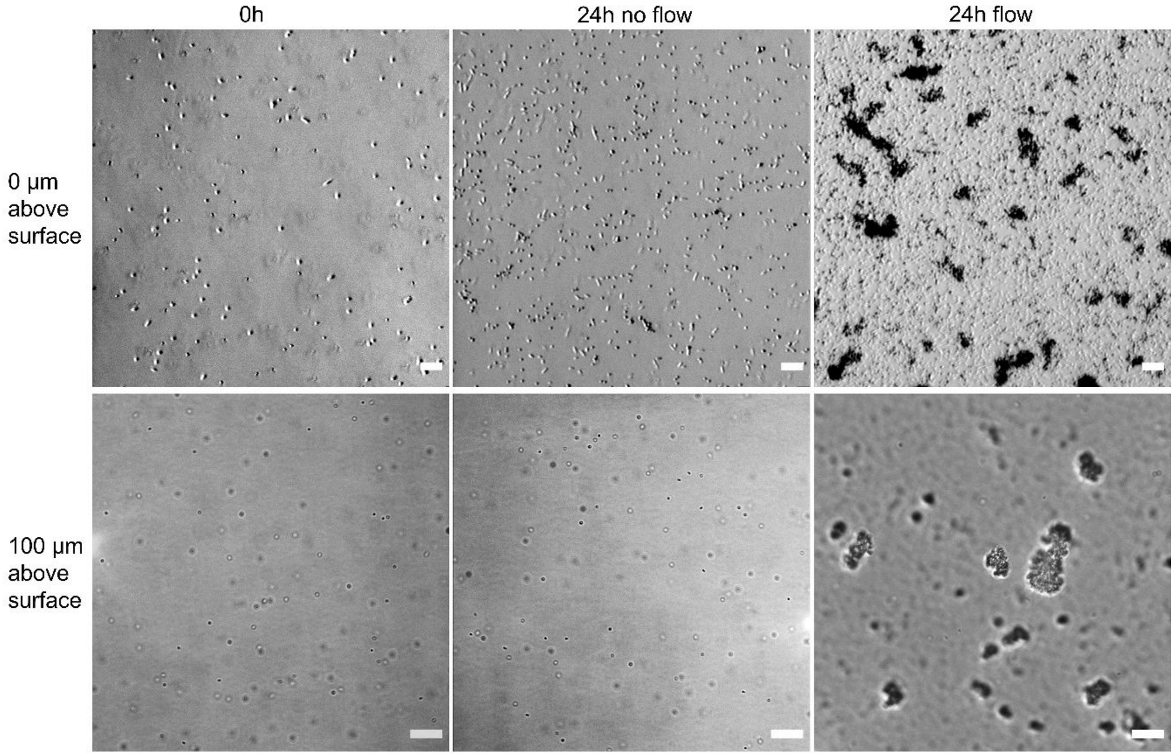

Supplementary figure 4. Representative differential interference contrast (DIC) microscopy images showing the lack of MPs+PA cells aggregation after 24h incubation under truly static conditions in <1 mm closed glass chambers (no flow) compared to mixtures incubated in glass-bottom dishes with medium currents due to convection (flow). Images were taken at 0 and 100 µm high from the centre of containers' bottom glass. Scale bar 10 µm.
