# Supplementary material for: Fluid dynamics and cell‐bound Psl polysaccharide allows microplastic capture, aggregation and subsequent sedimentation by Pseudomonas aeruginosa in water
Source: Environ Microbiol. 2022 Feb 2;24(3):1560–72. doi: 10.1111/1462-2920.15916 (PMC9305584; doi:10.1111/1462-2920.15916)
Supplement: Supplementary file 2 — Appendix S1: Supporting Information. [file EMI-24-1560-s002.pptx]

## Slide 1
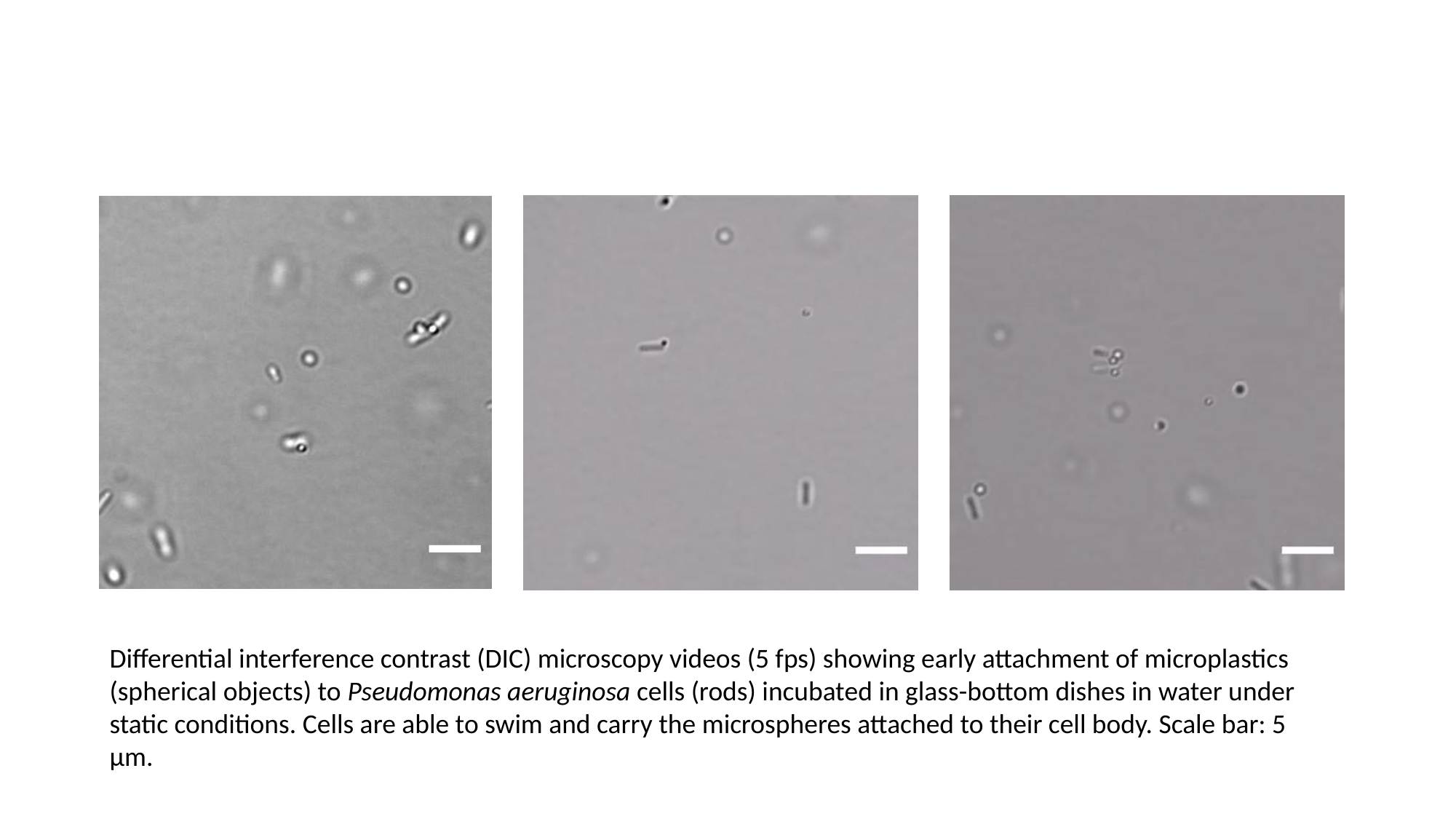

Differential interference contrast (DIC) microscopy videos (5 fps) showing early attachment of microplastics (spherical objects) to Pseudomonas aeruginosa cells (rods) incubated in glass-bottom dishes in water under static conditions. Cells are able to swim and carry the microspheres attached to their cell body. Scale bar: 5 µm.
